# ﻿Species of *Diaporthe* (Diaporthaceae, Diaporthales) associated with *Alnusnepalensis* leaf spot and branch canker diseases in Xizang, China

**DOI:** 10.3897/mycokeys.116.142750

**Published:** 2025-04-23

**Authors:** Jieting Li, Yi Li, Jiangrong Li, Ning Jiang

**Affiliations:** 1 Institute of Xizang Plateau Ecology, Key Laboratory of Forest Ecology in Xizang Plateau (Xizang Agricultural and Animal Husbandry University), Ministry of Education, Linzhi, Xizang 860000, China Xizang Agricultural and Animal Husbandry University Linzhi China; 2 National Forest Ecosystem Observation & Research Station of Linzhi Xizang, Linzhi, Xizang 860000, China National Forest Ecosystem Observation & Research Station of Linzhi Xizang Linzhi China; 3 Key Laboratory of Forest Protection of National Forestry and Grassland Administration, Ecology and Nature Conservation Institute, Chinese Academy of Forestry, Beijing 100091, China Ecology and Nature Conservation Institute, Chinese Academy of Forestry Beijing China

**Keywords:** Alder, molecular phylogeny, novel taxa, plant disease, Sordariomycetes, taxonomy

## Abstract

*Alnusnepalensis* is an important tree species in the Himalayas with significant ecological and economic roles. During disease surveys in Xizang, China, we observed leaf spot and branch canker symptoms on this tree. Fungal isolates associated with these diseases were collected and identified based on morphological characteristics and phylogenetic analysis of ITS, *cal*, *his3*, *tef1*, and *tub2* sequences. As a result, *Diaporthealnicola***sp. nov.** and *D.amygdali* were identified from the leaf spots, while *D.linzhiensis* was identified to be associated with the cankered branches. This study identifies pathogenic species from alder trees, providing a foundation for future disease management and forest health research.

## ﻿Introduction

*Alnusnepalensis* (Nepalese alder) is a tree species of significant ecological and economic importance, particularly in the temperate and subtropical regions of the Himalayas, including Xizang, Nepal, and northern India (Sharma et al. 1998; Xia et al. 2023). This plant fulfills a crucial role in maintaining the ecological balance of forest ecosystems (Tobita et al. 2016; Sen et al. 2022). Beyond its ecological importance, *A.nepalensis* also has considerable economic value (Saxena et al. 2016). Given its critical role in both ecosystem function and local economies, any threats to *A.nepalensis* populations, such as leaf spot and canker diseases, could have severe consequences for forest health.

*Diaporthe* is a pathogenic fungal genus in the Diaporthaceae (Diaporthales, Sordariomycetes, Ascomycota) (Udayanga et al. 2012; Dissanayake et al. 2017; Jiang et al. 2025). Species of this genus are commonly associated with plant diseases, acting as pathogens, endophytes, or saprobes (Dissanayake et al. 2020; Dong et al. 2021; Jiang et al. 2021). For example, *D.hsinchuensis* and five other species have been shown to cause leaf spots on *Camelliasinensis* (Ariyawansa et al. 2021). Several other *Diaporthe* species were shown to be associated with branch canker, dieback, and stem blight diseases (Guarnaccia et al. 2020; Guo et al. 2020; Bai et al. 2023). Additionally, *D.biconispora* and 15 other species from this genus were described as endophytes in *Citrus* (Huang et al. 2015).

*Diaporthe* is a species-rich genus with nearly 1,300 epithets listed in Index Fungorum (https://www.indexfungorum.org/). Over the past decade, many new species of this genus have been described based on both morphological characteristics and molecular phylogeny (Udayanga et al. 2014, 2015; Guarnaccia and Crous 2017; Yang et al. 2017, 2020, 2021; Fan et al. 2018; Manawasinghe et al. 2019; Huang et al. 2021; Sun et al. 2021; Cao et al. 2022; Lambert et al. 2023; Zhu et al. 2023, 2024; Liu et al. 2024). However, the species concepts of several taxa have been re-evaluated in recent years using the genealogical concordance phylogenetic species recognition (GCPSR) principle and coalescence-based models, such as the General Mixed Yule-Coalescent (GMYC) and Poisson Tree Processes (PTP). These re-evaluations have led to the synonymization of several species (Hilário et al. 2021a, 2021b; Dissanayake et al. 2024). For example, recent studies demonstrated that what was once thought to be a complex of nine species (*Diaportheamygdali* species complex) is actually a single species (Hilário et al. 2021a, 2021b).

Dissanayake et al. (2024) divided the genus *Diaporthe* into seven sections and 15 species complexes based on phylogenetic analysis of all available type isolates of this genus, such as section Rudis and the *D.virgiliae* species complex. This classification has simplified phylogenetic analysis during species identification. In the present study, a survey of alder diseases was conducted in Xizang, China, with the aim of identifying the fungal species associated with leaf spots and branch cankers through a combination of morphological and molecular approaches.

## ﻿Materials and methods

### ﻿Sample collection, isolation, and morphology

**Figure 1.**
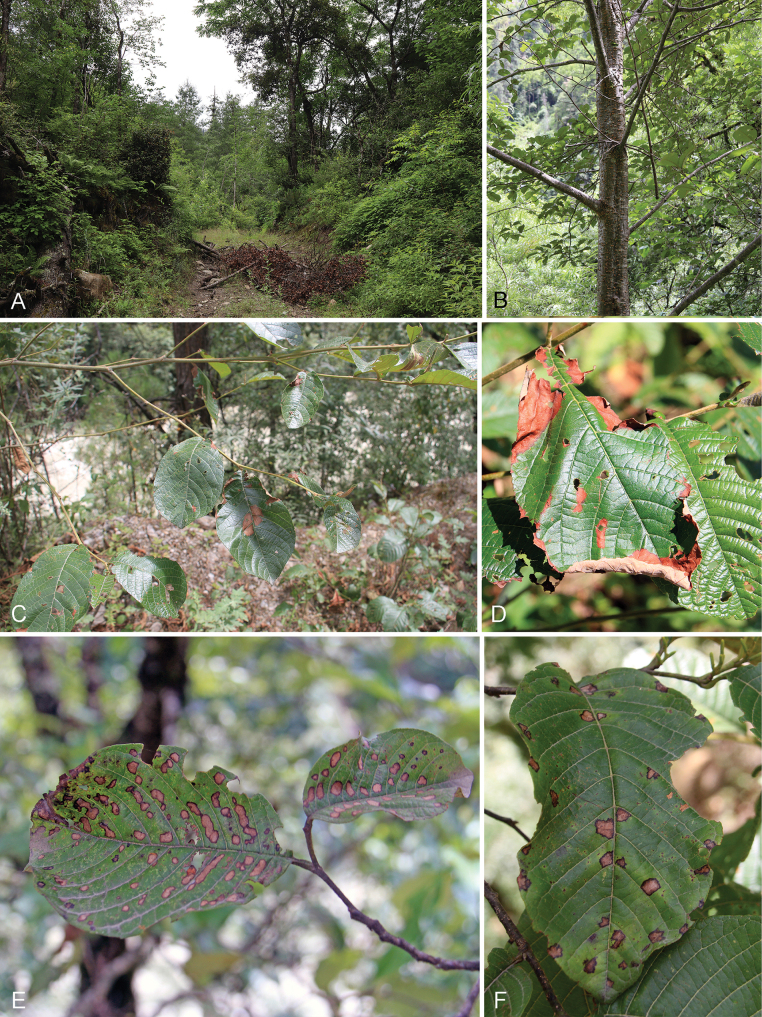
**A, B** sampling site **C–F** leaf spot symptoms of *Alnusnepalensis*.

Disease investigations were conducted from June to October in 2024 in Bayi District and Bomi County, Linzhi City, Xizang, China. Branch canker and leaf spot symptoms were observed, with canker being relatively rare and leaf spots more commonly encountered (Fig. 1). Infected branches exhibited sunken, discolored lesions, along with the presence of conidiomata of the fungal pathogen. Diseased leaves displayed small, rounded, or irregularly shaped spots, characterized by dark brown margins. Branch and leaf samples were collected and placed in paper envelopes for further analysis.

Sample branches and leaves were washed with sterile water and dried using refined absorbent cotton. Tissue fragments (5 × 5 mm) from both healthy and diseased samples were cut with a sterilized surgical knife, then immersed in 75% alcohol for 1 min, subsequently washed three times for 30 seconds each in sterile water, and dried with refined absorbent cotton. These tissue fragments were then transferred to the surface of Potato Dextrose Agar (PDA) plates. Hyphal tips grown from the tissue fragments on PDA were observed under a stereomicroscope (Discovery v8, Zeiss, Oberkochen, Germany). The fragments were then subcultured onto fresh PDA plates to obtain pure cultures. Type specimens were deposited in the herbarium of the Chinese Academy of Forestry (CAF), and ex-type isolates were stored in the China Forestry Culture Collection Center (CFCC, https://cfcc.caf.ac.cn/).

Cultures were grown on PDA, malt extract agar (MEA), and synthetic nutrient agar (SNA) plates for observation. Conidiomata formed on the culture plates and branches were studied. The conidiomata were carefully sectioned using a double-edged blade, and fungal structures were observed under a Zeiss Discovery v8 stereomicroscope. Conidiophores, conidiogenous cells, and conidia were further examined and photographed using an Olympus BX51 microscope (Tokyo, Japan).

### ﻿Phylogenetic analyses

The genomic DNA of the *Diaporthe* isolates obtained in this study was extracted from young colonies grown on PDA plates following the protocol of Doyle and Doyle (1990). The internal transcribed spacer (ITS) region of rDNA, along with fragments of the calmodulin (*cal*), histone H3 (*his3*), translation elongation factor 1-alpha (*tef1*), and partial beta-tubulin (*tub2*) genes, was amplified using the primers and protocols outlined in Table 1. The PCR products were subjected to electrophoresis on 2% agarose gels for analysis, followed by sequencing using the same primers as those employed in the PCR amplification. The sequencing service was provided by Ruibo Xingke Biotechnology Co., Ltd. (Beijing, China).

**Table 1.** Primers and PCR protocols.

Gene Regions	Primers	PCR conditions	References
ITS	ITS1/ITS4	95 °C for 4 min, 35 cycles of 94 °C for 45 s, 48 °C for 1 min, and 72 °C for 2 min, 72 °C for 10 min	White et al. 1990
*cal*	CAL228F/CAL737R	95 °C for 4 min, 35 cycles of 94 °C for 45 s, 54 °C for 1 min, and 72 °C for 2 min, 72 °C for 10 min	Carbone and Kohn 1999
*his3*	CYLH3F/H3-1b	95 °C for 5 min, 35 cycles of 95 °C for 1 min, 57 °C, 1.25 min, and 72 °C for 2 min, 72 °C for 10 min	Crous et al. 2004; Glass and Donaldson 1995
*tef1*	EF1-728F/EF1-986R	94 °C for 3 min, 35 cycles of 94 °C for 30 s, 54 °C for 50 s, and 72 °C for 2 min, 72 °C for 10 min	Carbone and Kohn 1999
*tub2*	T1(Bt2a)/Bt2b	95 °C for 4 min, 35 cycles of 94 °C for 45 s, 54 °C for 1 min, and 72 °C for 2 min, 72 °C for 10 min	Glass and Donaldson 1995; O’Donnell and Cigelnik 1997

The ITS, *cal*, *his3*, *tef1*, and *tub2* gene sequences obtained in this study were queried against the GenBank nucleotide database located at the National Center for Biotechnology Information (NCBI) to identify closely related sequences and determine the associated species. Sequence data for related taxa were retrieved from Dissanayake et al. (2024) and downloaded from NCBI (Table 2). The sequences were aligned using the MAFFT v.7 online server (http://mafft.cbrc.jp/alignment/server/index.html, Katoh et al. 2019) with default settings.

**Table 2.** GenBank accession numbers used in the phylogenetic analyses.

Species	Strain	GenBank accession numbers					References
		ITS	*tef1*	*tub2*	*cal*	*his3*	
*Diaportheacaciigena*	CBS 129521	KC343005	KC343731	KC343973	KC343247	KC343489	Gomes et al. 2013
***D.alnicola***	CFCC 70997*	**PQ636515**	**PQ635059**	**PQ635065**	**PQ635047**	**PQ635053**	**In this study**
***D.alnicola***	CFCC 70998*	**PQ636516**	**PQ635060**	**PQ635066**	**PQ635048**	**PQ635054**	**In this study**
*D.amygdali*	CBS 126679	KC343022	KC343748	KC343990	KC343264	KC343506	Gomes et al. 2013
*D.amygdali*	CBS 111811	KC343019	KC343745	KC343987	KC343261	KC343503	Gomes et al. 2013
*D.amygdali*	CBS 115620	KC343020	KC343746	KC343988	KC343262	KC343504	Gomes et al. 2013
*D.amygdali*	CBS 120840	KC343021	KC343747	KC343989	KC343263	KC343505	Gomes et al. 2013
*D.amygdali* syn. *D.chongqingensis*	CGMCC 3.19603	MK626916	MK654866	MK691321	MK691209	MK726257	Guo et al. 2020
*D.amygdali* syn. *D.chongqingensis*	PSCG 435	MK626916	MK654866	MK691321	MK691209	MK726257	Guo et al. 2020
*D.amygdali* syn. *D.chongqingensis*	PSCG 436	MK626917	MK654867	MK691322	MK691208	MK726256	Guo et al. 2020
*D.amygdali* syn. *D.chongqingensis*	PSCG 436-2	MK626917	MK654867	MK691322	MK691208	MK726256	Guo et al. 2020
*D.amygdali* syn. *D.fusicola*	CGMCC 3.17087	KF576281	KF576256	KF576305	KF576233	NA	Gao et al. 2015
*D.amygdali* syn. *D.fusicola*	CGMCC 3.17088	KF576263	KF576238	KF576287	KF576221	NA	Gao et al. 2015
*D.amygdali* syn. *D.garethjonesii*	MFLUCC 12-0542	KT459423	KT459457	KT459441	KT459470	NA	Gao et al. 2015
*D.amygdali* syn. *D.kadsurae*	CFCC 52586	MH121521	MH121563	MH121600	MH121439	MH121479	Yang et al. 2018
*D.amygdali* syn. *D.kadsurae*	CFCC 52587	MH121522	MH121564	MH121601	MH121440	MH121480	Yang et al. 2018
*D.amygdali* syn. *D.mediterranea*	CBS 146754	MT007496	MT006996	MT006693	MT006768	MT007102	León et al. 2020
*D.amygdali* syn. *D.ovoicicola*	CGMCC 3.17092	KF576264	KF576239	KF576288	KF576222	NA	Gao et al. 2015
*D.amygdali* syn. *D.ovoicicola*	CGMCC 3.17093	KF576265	KF576240	KF576289	KF576223	NA	Gao et al. 2015
*D.amygdali* syn. *D.ovoicicola*	CGMCC 3.17094	KF576266	KF576241	KF576290	KF576224	NA	Gao et al. 2015
*D.amygdali* syn. *D.ovoicicola*	ACJY62	MW578711	MW597404	MW598141	MW598161	MW598183	Gao et al. 2015
*D.amygdali* syn. *D.sterilis*	CBS 136969	KJ160579	KJ160611	KJ160528	KJ160548	MF418350	Lombard et al. 2014
*D.amygdali* syn. *D.sterilis*	CPC 20580	KJ160582	KJ160614	KJ160531	KJ160551	NA	Lombard et al. 2014
*D.amygdali* syn. *D.ternstroemia*	CGMCC 3.15183	KC153098	KC153089	NA	NA	NA	Gao et al. 2014
*D.amygdali* syn. *D.ternstroemia*	CGMCC 3.15184	KC153099	KC153090	NA	NA	NA	Gao et al. 2014
***D.amygdali***	**CFCC 70999**	**PQ636517**	**PQ635061**	**PQ635067**	**PQ635049**	**PQ635055**	**In this study**
***D.amygdali***	**Q3B**	**PQ636518**	**PQ635062**	**PQ635068**	**PQ635050**	**PQ635056**	**In this study**
*D.araucanorum*	CBS 145285	MN509711	MN509733	MN509722	NA	NA	Zapata et al. 2020
*D.araucanorum*	CBS 145283	MN509709	MN509731	MN509720	NA	NA	Zapata et al. 2020
*D.beckhausii*	CBS 138.27	KC343041	KC343767	KC344009	KC343283	KC343525	Gomes et al. 2013
*D.benedicti*	BPI 893190	KM669929	KM669785	NA	KM669862		Lawrence et al. 2015
*D.breviconidiophora*	CGMCC 3.24298	OP056725	OP150564	OP150641	OP150718	OP150794	Dissanayake et al. 2024
*D.breviconidiophora*	GZCC 22-0030	OP056725	OP150564	OP150641	OP150718	OP150794	Dissanayake et al. 2024
*D.cassines*	CPC 21916	KF777155	KF777244	NA	NA	NA	Crous et al. 2013
*D.celticola*	CFCC 53074	MK573948	MK574623	MK574643	MK574587	MK574603	Cao et al. 2022
*D.celticola*	CFCC 53075	MK573949	MK574624	MK574644	MK574588	MK574604	Cao et al. 2022
*D.crousii*	CAA823	MK792311	MK828081	MK837932	MK883835	MK871450	Hilário et al. 2020
*D.crousii*	CAA820	MK792300	MK828072	MK837923	MK883828	MK871441	Hilário et al. 2020
*D.eres*	AR5193	KJ210529	KJ210550	KJ420799	KJ434999	KJ420850	Udayanga et al. 2014
*D.eres*	DLR12a	KJ210518	KJ210542	KJ420783	KJ434996	KJ420833	Udayanga et al. 2014
*D.foikelawen*	CBS 145289	MN509713	MN509735	MN509724	NA	NA	Zapata et al. 2020
*D.foikelawen*	CBS 145287	MN509714	MN509736	MN509725	NA	NA	Zapata et al. 2020
*D.grandifori*	SAUCC 194.84	MT822612	MT855924	MT855809	MT855691	MT855580	Sun et al. 2021
*D.guizhouensis*	GZCC 20-0338	OM060254	OL961761	OL961762	OL961763	NA	Bhunjun et al. 2022
*D.guizhouensis*	GZCC 22-0027	OP056683	OP150522	OP150600	OP150679	OP150754	Bhunjun et al. 2022
*D.guizhouensis*	GZCC 22-0045	OP056684	OP150523	OP150601	OP150680	OP150755	Bhunjun et al. 2022
*D.heterophyllae*	CBS 143769	MG600222	MG600224	MG600226	MG600218	MG600220	Marín-Felix et al. 2019
*D.heveae*	B23	KR812219	NA	NA	NA	NA	Dos Santos et al. 2015
***D.linzhiensis***	CFCC 71057*	**PQ636519**	**PQ635063**	**PQ635069**	**PQ635051**	**PQ635057**	**In this study**
***D.linzhiensis***	N266C*	**PQ636520**	**PQ635064**	**PQ635070**	**PQ635052**	**PQ635058**	**In this study**
*D.nothofagi*	BRIP 54801	JX862530	JX862536	KF170922	NA	NA	Tan et al. 2013
*D.obtusifoliae*	CBS 143449	MG386072	NA	NA	NA	MG386137	Crous et al. 2017
*D.ocoteae*	CPC 26217	KX228293	NA	KX228388	NA	NA	Crous et al. 2013
*D.penetriteum*	LC3353	KP714505	KP714517	KP714529	NA	KP714493	Gao et al. 2016
*D.pustulata*	CBS 109742	KC343185	KC343911	KC344153	KC343427	KC343669	Gomes et al. 2013
*D.pustulata*	CBS 109784	KC343187	KC343913	KC344155	KC343429	KC343671	Gomes et al. 2013
*D.rudis*	AR3422	KC843331	KC843090	KC843177	KC843146	NA	Udayanga et al. 2014
*D.rudis*	AR3654	KC843338	KC843097	KC843184	KC843153	NA	Udayanga et al. 2014
*D.rudis*	DA244	KC843334	KC843093	KC843180	KC843149	NA	Udayanga et al. 2014
*D.rudis*	ICMP 16419	KC145904	KC145976	NA	NA	NA	Udayanga et al. 2014
*D.rudis*	ICMP 7025	KC145885	KC145995	NA	NA	NA	Udayanga et al. 2014
*D.rudis*	CBS 113201	MH862916	KC343960	KC344202	KC343476	KC343718	Vu et al. 2019
*D.rudis* syn. *D.australafricana*	CBS 111886	KC343038	KC343764	KC344006	KC343280	KC343522	Gomes et al. 2013
*D.rudis* syn. *D.australafricana*	CBS 113487	KC343039	KC343765	KC344007	KC343281	KC343523	Gomes et al. 2013
*D.rudis* syn. *D.cynaroidis*	CBS 122676	KC343058	KC343784	KC344026	KC343300	KC343542	Gomes et al. 2013
*D.rudis* syn. *D.patagonica*	CBS 145291	MN509717	MN509739	MN509728	NA	NA	Zapata et al. 2020
*D.rudis* syn. *D.patagonica*	CBS 145755	MN509718	MN509740	MN509729	NA	NA	Zapata et al. 2020
*D.rudis* syn. *D.salicicola*	BRIP 54825	JX862531	JX862537	KF170923	NA	NA	Tan et al. 2013
*D.rudis* syn. *D.subcylindrospora*	KUMCC 17-0151	MG746629	MG746630	MG746631	NA	NA	Hyde et al. 2018
*D.shennongjiaensis*	CNUCC 201905	MN216229	MN224672	MN227012	MN224551	MN224559	Zhou and Hou 2019
*D.shennongjiaensis*	CNUCC 201906	MN216228	MN224673	MN227013	MN224552	MN224561	Zhou and Hou 2019
*D.silvicola*	CFCC 54191	MZ727041	MZ816347	MZ753491	MZ753472	MZ753481	Jiang et al. 2021
*D.silvicola*	M79	MZ727042	MZ816348	MZ753492	MZ753473	MZ753482	Jiang et al. 2021
*D.torilicola*	MFLUCC 17-1051	KY964212	KY964168	KY964096	KY964127		Dissanayake et al. 2017
*D.toxica*	CBS 534.93	KC343220	KC343946	KC344188	KC343462	KC343704	Gomes et al. 2013
*D.toxica*	CBS 546.93	KC343222	KC343948	KC344190	KC343464	KC343706	Gomes et al. 2013
*D.virgiliae*	CMW 40755	KP247573	NA	KP247582	NA	NA	Machingambi et al. 2015
*D.virgiliae*	CMW 40748	KP247566	NA	KP247575	NA	NA	Machingambi et al. 2015
*D.zaofenghuang*	CGMCC 3.20271	MW477883	MW480871	MW480875	MW480867	MW480863	Wang et al. 2021
*D.zaofenghuang*	TZFH3	MW477884	MW480872	MW480876	MW480868	MW480864	Wang et al. 2021

**Note**: “NA” indicates unavailable sequences; sequences produced in the current study are in bold, and * means ex-type strains from new species in this study.

The isolates described in this study were shown to belong to the DiaportheSectionRudis and the D.virgiliae species complex, respectively. Maximum likelihood (ML) phylogenetic analysis was conducted using the CIPRES Science Gateway platform (Miller et al. 2010), with RAxMLHPC2 on XSEDE (v. 8.2.10) under the GTR substitution model and 1000 non-parametric bootstrap replicates. Bayesian analysis was performed with MrBayes v. 3.2.6, utilizing four simultaneous Markov chain runs for 1,000,000 generations. The resulting trees were visualized using FigTree v. 1.4.0 (Rambaut 2012).

The pairwise homoplasy index test was employed to confirm the new species status using SplitsTree v.4.16.1 (Huson and Bryant 2006). Incongruence among the ITS-*cal*-*his3*-*tef1*-*tub2* genealogies was used as a criterion to identify hypothesized “species” and infer the occurrence of sexual recombination (Bruen et al. 2006). Results of the Фw-statistic below a 0.05 threshold (*p*-value < 0.05) indicated significant recombination. A phylogenetic network based on the combined dataset of five loci was constructed using the NeighborNet algorithm to assess the impact of recombination.

## ﻿Results

### ﻿Phylogenetic analyses

**Figure 2.**
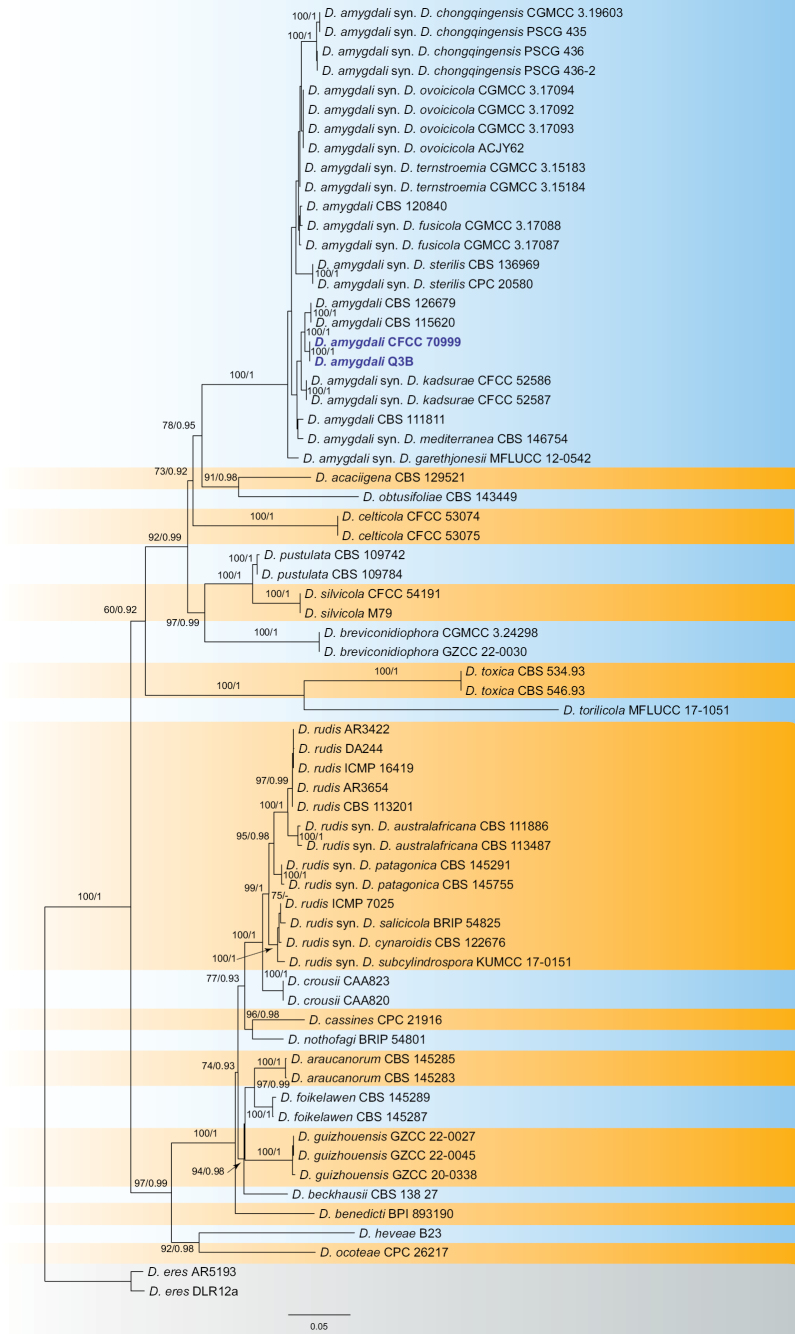
Maximum likelihood tree of DiaportheSectionRudis generated from combined ITS, *cal*, *his3*, *tef1*, and *tub2* sequence data. Bootstrap support values ≥ 50% and Bayesian posterior probabilities ≥ 0.90 are demonstrated at the branches. Isolates from the present study are indicated in blue.

For the analysis of DiaportheSectionRudis, the combined dataset of ITS, *cal*, *his3*, *tef1*, and *tub2* comprised 67 strains, with *D.eres* (AR5193 and DLR12a) used as the outgroup taxa. The final alignment included 2,691 characters (ITS: 451, *cal*: 702, *his3*: 410, *tef1*: 596, *tub2*: 532), including gaps. The final ML optimization likelihood value of the best RAxML tree was -17019.93, and the matrix contained 1,257 distinct alignment patterns, with 32.15% undetermined characters or gaps. The estimated base frequencies were A = 0.216951, C = 0.313266, G = 0.235799, T = 0.233984; substitution rates were AC = 1.028567, AG = 3.157223, AT = 1.223911, CG = 0.822997, CT = 4.362405, GT = 1.0; and the gamma distribution shape parameter α = 0.386104. Both the RAxML and Bayesian analyses produced similar tree topologies, which were consistent with those of previous studies (Norphanphoun et al. 2022; Dissanayake et al. 2024). Isolates from this study (CFCC 70999 and Q3B) clustered together with other *Diaportheamygdali* strains, showing strong support (Fig. 2), thus confirming their identification as *D.amygdali*.

In the *Diaporthevirgiliae* species complex, the combined dataset of ITS, *cal*, *his3*, *tef1*, and *tub2* included 13 strains, with *D.shennongjiaensis* (CUNCC 201905 and CUNCC 201906) as the outgroup taxa. The final alignment contained 2,598 characters (ITS: 593, *cal*: 421, *his3*: 466, *tef1*: 331, *tub2*: 787), including gaps. The final ML optimization likelihood value of the best RAxML tree was -5834.44, and the matrix had 346 distinct alignment patterns, with 20.11% undetermined characters or gaps. The estimated base frequencies were A = 0.212455, C = 0.329026, G = 0.238268, T = 0.220251; substitution rates were AC = 1.111868, AG = 2.843163, AT = 1.775735, CG = 0.816784, CT = 3.662621, GT = 1.0; and the gamma distribution shape parameter α = 0.047755. Both RAxML and Bayesian analyses produced similar tree topologies, which closely matched those of prior publications (Norphanphoun et al. 2022; Dissanayake et al. 2024). Four isolates from this study formed two new clades distinct from any lineage and are hence accommodated as two novel species: *D.alnicola* (CFCC 70997 and CFCC 70998) and *D.linzhiensis* (CFCC 71057 and N266C).

The network relationships within the *D.virgiliae* species complex are depicted in Fig. 4, indicating no significant recombination based on the PHI test (*p* = 0.9624). Furthermore, based on the relative distances between species and the structure of the phylogenetic network, isolates within the *D.virgiliae* complex represent seven different species.

### ﻿Taxonomy

#### ﻿ Diaporthe alnicola

Taxon classificationFungiDiaporthalesDiaporthaceae

Ning Jiang sp. nov.

C91816DF-5FCC-516B-AD00-5AA56EFC8584

856742

Fig. 5

##### Etymology.

“*Alni*” refers to the host genus *Alnus*, and “-*cola*” means inhabiting.

##### Description.

Associated with leaf spot disease of *Alnusnepalensis*. ***Teleomorph***: Undetermined. ***Anamorph***: Conidiomata formed on PDA pycnidial, scattered, erumpent, pulvinate to subglobose, dark brown, 150–350 μm diam. Conidiophores indistinct, usually reduced to conidiogenous cells. Conidiogenous cells cylindrical, attenuate towards the apex, hyaline, phialidic, 9.5–33 × 2–3 μm. Alpha conidia aseptate, hyaline, smooth, guttulate, cylindrical, straight, base truncate, (6–)6.5–7(–7.5) × (2–)2.5–3(–3.5) μm (x̄ = 6.8 × 2.6 μm, n = 50), L/W = 2–3.4. Beta conidia aseptate, hyaline, smooth, guttulate, filiform, tapering towards both ends, curved, (13–)14.5–22(–24) × 1.5–2.5 μm (x̄ = 18.3 × 2.1 μm, n = 50), L/W = 5.9–12.5. Gamma conidia not observed.

##### Culture characteristics.

Colonies on PDA at 25 °C are spreading, flocculent, forming abundant aerial mycelium and an undulate margin, initially white, turning mouse gray and reaching a diameter of 90 mm after 10 d, developing dark brown conidiomata with orange conidial masses after 20 d. Colonies on MEA at 25 °C are flat, spreading, feathery, with a smooth entire margin, white, reaching a diameter of 90 mm after 15 d, sterile. Colonies on SNA at 25 °C are flat, spreading with a smooth entire margin, white, reaching 90 mm in diameter after 20 d, developing dark brown conidiomata with orange conidial masses after 30 d.

##### Materials examined.

China • Xizang Autonomous Region (Tibet), Linzhi City, Bayi District, Pailong Town, 30°4'22"N, 95°8'2"E, 2192 m, from leaf spots of *Alnusnepalensis*, 9 Jul. 2024, *Ning Jiang, Jieting Li & Haoyin Zhang* (***holotype*** CAF800100, ex-paratype cultures CFCC 70997 and CFCC 70998).

##### Notes.

**Figure 3.**
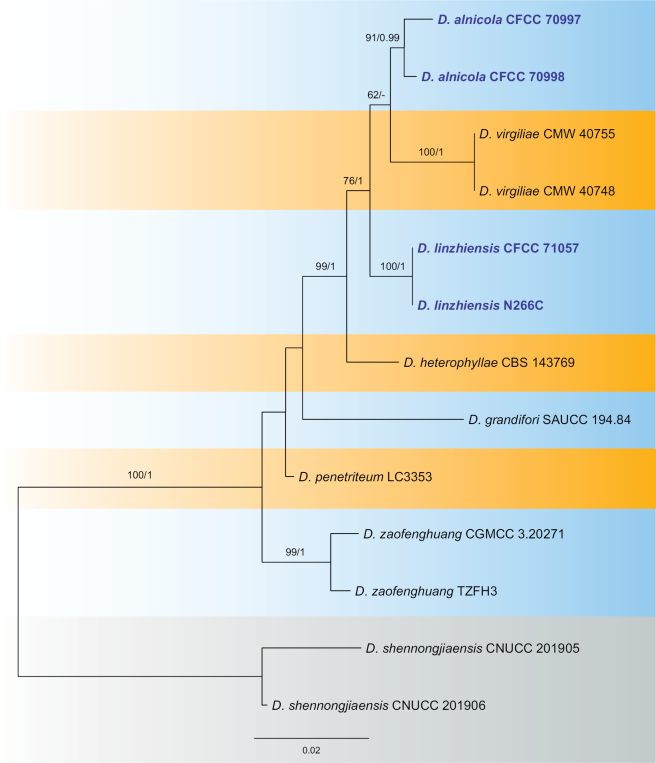
Maximum likelihood tree of the *Diaporthevirgiliae* species complex generated from combined ITS, *cal*, *his3*, *tef1*, and *tub2* sequence data. Bootstrap support values ≥ 50% and Bayesian posterior probabilities ≥ 0.90 are demonstrated at the branches. Isolates from the present study are indicated in blue.

*Diaporthealnicola*, identified from leaf spots on *Alnusnepalensis* in this study, is phylogenetically closely related to *D.virgiliae*, which originates from the rot root of *Virgiliaoroboides* in South Africa (Fig. 3). Morphologically, *D.alnicola* is similar to *D.virgiliae* in terms of the size of alpha and beta conidia (alpha conidia: 6.5–7 × 2.5–3 μm in *D.alnicola* vs. 5.2–8 × 1.1–3.5 μm in *D.virgiliae*; beta conidia: 14.5–22 × 1.5–2.5 μm in *D.alnicola* vs. 17.1–25.4 × 1–1.8 μm in *D.virgiliae*). However, they can be distinguished by the size of their conidiogenous cells (9.5–33 × 2–3 μm in *D.alnicola* vs. 12.3–21.3 × 0.7–1.5 μm in *D.virgiliae*) (Machingambi et al. 2015). Furthermore, *D.alnicola* differs from *D.virgiliae* at the nucleotide level (ITS, 11/432; *tub2*, 7/743).

**Figure 4.**
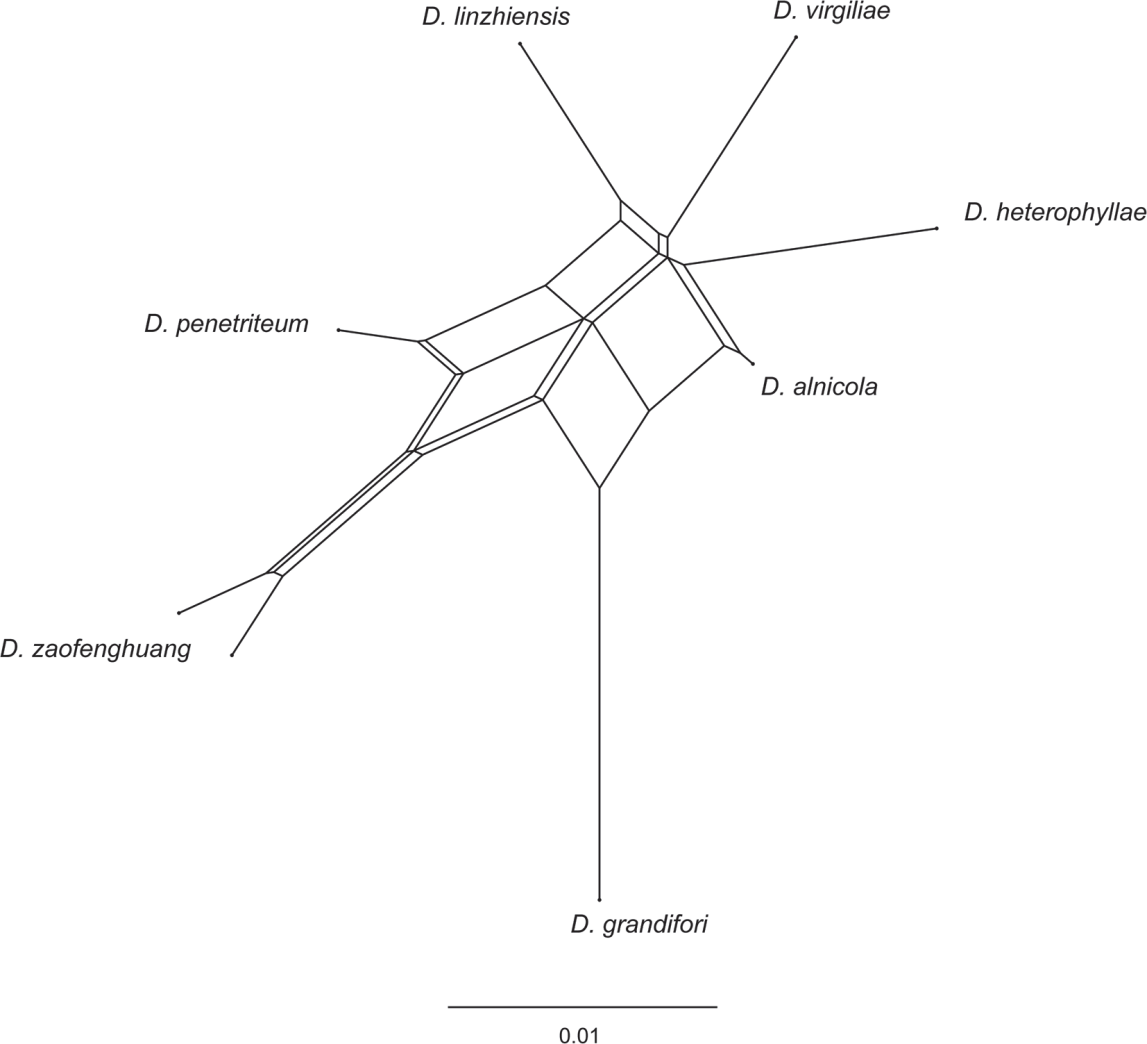
Phylogenetic network from concatenated data (ITS, *cal*, *his3*, *tef1*, and *tub2*) representing the structure of the *Diaporthevirgiliae* species complex, based on LogDet transformation and the NeighborNet algorithm, inferred by SplitsTree (*p* = 0.9624). The scale bar represents the expected number of substitutions per nucleotide position.

**Figure 5.**
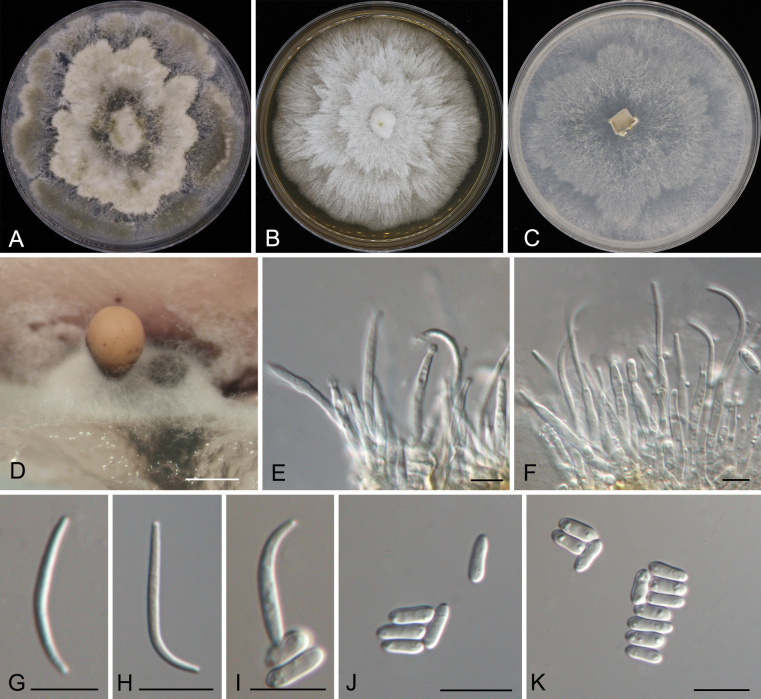
Morphology of *Diaporthealnicola***A** colony on PDA after 15 d **B** Colony on MEA after 15 d **C** colony on SNA after 15 d **D** conidioma formed on PDA**E, F** conidiogenous cells **G–K** alpha and beta conidia. Scale bars: 500 µm (**D**); 10 µm (**E–K**).

#### ﻿ Diaporthe amygdali

Taxon classificationFungiDiaporthalesDiaporthaceae

(Delacr.) Udayanga, Crous & K.D. Hyde, Fungal Diversity 56(1): 166. 2012

E293E841-E0FD-58C9-B929-DC5D121F5CA8

Fig. 6

##### Description.

Associated with leaf spot disease of *Alnusnepalensis*. ***Teleomorph***: Undetermined. ***Anamorph***: Conidiomata formed on PDA pycnidial, scattered, erumpent, subglobose, dark brown, 700–2250 μm diam. Conidiophores indistinct, usually reduced to conidiogenous cells. Conidiogenous cells cylindrical, attenuate towards the apex, hyaline, phialidic, 16.5–34 × 1.5–3 μm. Alpha conidia not observed. Beta conidia aseptate, hyaline, smooth, guttulate, filiform, tapering towards both ends, straight or slightly curved, (27.5–)30–35(–40.5) × 1.5–2 μm (x̄ = 32.6 × 1.6 μm, n = 50), L/W = 15.8–23.1. Gamma conidia not observed.

**Figure 6.**
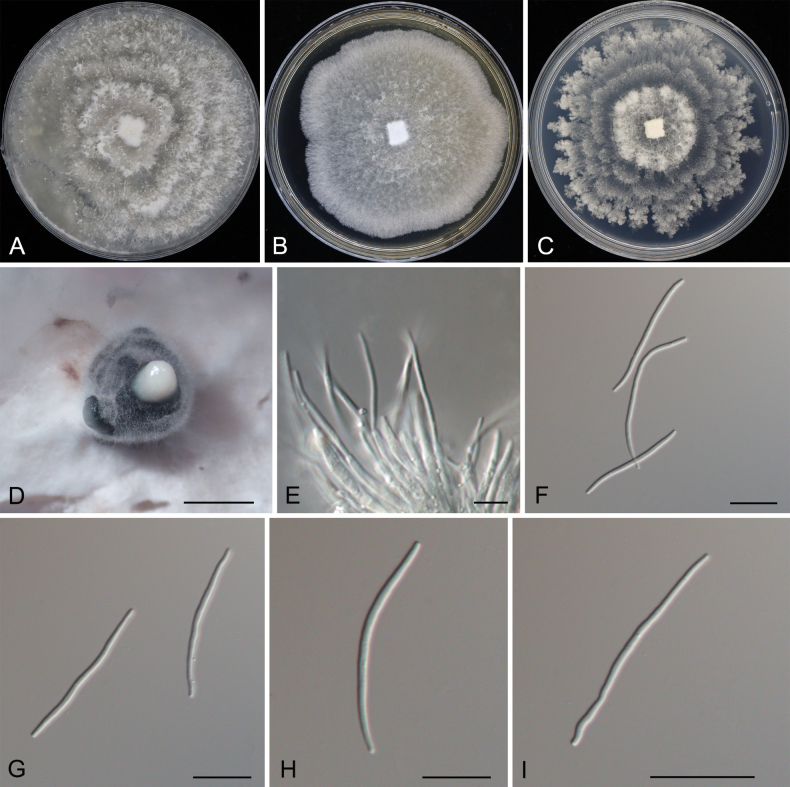
Morphology of *Diaportheamygdali***A** colony on PDA after 15 d **B** colony on MEA after 15 d **C** colony on SNA after 15 d **D** conidioma formed on PDA**E** conidiogenous cells **E–I** beta conidia. Scale bars: 800 µm (**D**); 10 µm (**E–K**).

##### Culture characteristics.

Colonies on PDA at 25 °C are flocculent, forming concentric zones with undulate margins, initially white, turning pale brownish, and reaching a diameter of 90 mm after 10 d, developing dark brown conidiomata with white conidial masses after 25 d. Colonies on MEA at 25 °C are flat, spreading, with a smooth entire margin, white, reaching a diameter of 80 mm after 20 d, sterile. Colonies on SNA at 25 °C are flat, spreading with a feathery margin, white, reaching 80 mm in diameter after 20 d, sterile.

##### Materials examined.

China • Xizang Autonomous Region (Tibet), Linzhi City, Bayi District, Pailong Town, 30°4'22"N, 95°8'2"E, 2192 m, from leaf spots of *Alnusnepalensis*, 9 Jul. 2024, *Ning Jiang, Jieting Li & Haoyin Zhang* (cultures CFCC 70999 and Q3B).

##### Notes.

The species concept of *Diaportheamygdali* has been revised in recent studies using phylogenetic analysis, GCPSR, and coalescence-based models (Hilário et al. 2021b; Dissanayake et al. 2024). Currently, *D.amygdali* is considered synonymous with *D.chongqingensis*, *D.fusicola*, *D.garethjonesii*, *D.kadsurae*, *D.mediterranea*, *D.ovoicicola*, *D.sterilis*, and *D.ternstroemia* (Hilário et al. 2021b; Dissanayake et al. 2024). This fungus is widely distributed, inhabiting a range of plant hosts, including *Acer* spp., *Camelliasinensis*, *Lithocarpusglabra*, *Prunusdulcis*, *Prunuspersica*, *Prunussalicina*, *Pyruspyrifolia*, *Ternstroemiagymnanthera*, *Vacciniumcorymbosum*, and *Vitisvinifera* (Hilário et al. 2021b). In this study, two isolates from leaf spots of *Alnusnepalensis* clustered with strains of *D.amygdali* with high support values (Fig. 2). Therefore, these two isolates were identified as *D.amygdali*, which led us to describe *Alnusnepalensis* as a new host for this fungus.

#### ﻿ Diaporthe linzhiensis

Taxon classificationFungiDiaporthalesDiaporthaceae

Ning Jiang sp. nov.

BADA5B89-517D-5F75-94FC-374040BB8070

856743

Fig. 7

##### Etymology.

Named after the collection site of the type specimen, Linzhi City.

**Figure 7.**
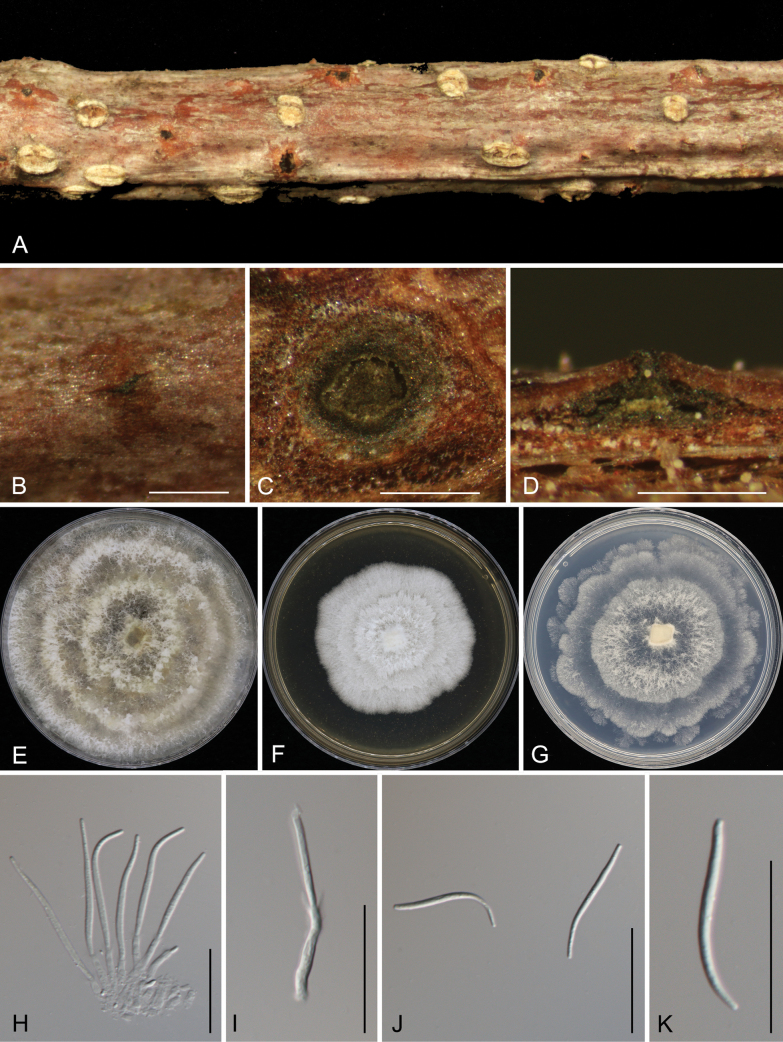
Morphology of *Diaporthelinzhiensis***A, B** conidiomata formed on twigs of *Alnusnepalensis***C** transverse section through a conidioma **D** longitudinal section through a conidioma **E** colony on PDA after 15 d **F** colony on MEA after 15 d **G** colony on SNA after 15 d **H, I** conidiogenous cells **J, K** beta conidia. Scale bars: 500 µm (**B–D**); 20 µm (**H–K**).

##### Description.

Associated with branch canker disease of *Alnusnepalensis*. ***Teleomorph***: Undetermined. ***Anamorph***: Conidiomata pycnidial, immersed in bark, scattered, erumpent through the bark surface, conical, with a solitary locule, 300–500 μm diam., 250–400 μm high. Conidiophores reduced to conidiogenous cells. Conidiogenous cells cylindrical, attenuate towards the apex, hyaline, phialidic, straight or slightly curved, 5.5–16 × 1.5–3 μm. Alpha conidia not observed. Beta conidia aseptate, hyaline, smooth, guttulate, filiform, tapering towards both ends, straight or slightly curved, (23.5–)24.5–29(–30) × 1.5–2 μm (x̄ = 26.6 × 1.8 μm, n = 50), L/W = 12.4–19.4. Gamma conidia not observed.

##### Culture characteristics.

Colonies on PDA at 25 °C are spreading, flocculent, forming abundant aerial mycelium and concentric zones with an undulate margin, initially white, turning pale luteous, and reaching a diameter of 90 mm after 10 d, sterile. Colonies on MEA at 25 °C are flat, spreading, with a smooth entire margin, white, reaching a diameter of 60 mm after 20 d, sterile. Colonies on SNA at 25 °C are flat, spreading, forming concentric zones with undulate margins, white, reaching 80 mm in diameter after 20 d, sterile.

##### Materials examined.

China • Xizang Autonomous Region (Tibet), Linzhi City, Bomi County, Tongmai Town, 30°5'53"N, 95°3'49"E, 2055 m, from branches of *Alnusnepalensis*, 9 Jul. 2024, *Ning Jiang, Jieting Li & Haoyin Zhang* (***holotype*** CAF800101, ex-paratype cultures CFCC 71057 and N266C).

##### Notes.

*Diaporthelinzhiensis* is phylogenetically closely related to *D.alnicola*, *D.heterophyllae*, and *D.virgiliae* (Fig. 2). Both *D.linzhiensis* and *D.alnicola* infect *Alnusnepalensis* in China, while *D.heterophyllae* is found on *Acaciaheterophylla* in France, and *D.virgiliae* inhabits *Virgiliaoroboides* in South Africa (Machingambi et al. 2015; Marín-Felix et al. 2019). Morphologically, *D.linzhiensis* shares a similar conidiogenous cell size with *D.alnicola* and *D.heterophyllae*, which is wider than that of *D.virgiliae* (5.5–16 × 1.5–3 μm in *D.linzhiensis* vs. 9.5–33 × 2–3 μm in *D.alnicola* vs. 6–9 × 1–2 μm in *D.heterophyllae* vs. 12.3–21.3 × 0.7–1.5 μm in *D.virgiliae*). Additionally, *D.linzhiensis* has longer beta conidia compared to the other species (24.5–29 × 1.5–2 μm in *D.linzhiensis* vs. 14.5–22 × 1.5–2.5 μm in *D.alnicola* vs. 17–24 × 1–2 μm in *D.heterophyllae* vs. 17.1–25.4 × 1–1.8 μm in *D.virgiliae*) (Machingambi et al. 2015; Marín-Felix et al. 2019). At the nucleotide level, *D.linzhiensis* also differs from *D.alnicola* (ITS, 23/547; *cal*, 2/382; *his3*, 6/469; *tef1*, 4/349; *tub2*, 6/778), *D.heterophyllae* (ITS, 28/560; *cal*, 11/420; *his3*, 6/447; *tef1*, 12/326; *tub2*, 3/406), and *D.virgiliae* (ITS, 16/434; *tub2*, 7/743).

## ﻿Discussion

This study enhances the understanding of *Diaporthe* species on alder by revealing two previously undescribed species and a new host association, viz. *Diaporthealnicola* sp. nov., *D.linzhiensis* sp. nov., and *D.amygdali* on *Alnusnepalensis. Diaporthe* is a morphologically distinct genus characterized by the production of alpha, beta, and gamma conidia. The alpha conidia are typically aseptate, hyaline, guttulate, and cylindrical to fusiform, while the beta conidia are aseptate, hyaline, and filiform (Farr et al. 2002; Udayanga et al. 2015; Guarnaccia and Crous 2017; Manawasinghe et al. 2019; Huang et al. 2021; Sun et al. 2021; Lambert et al. 2023). However, species within the genus usually share the same host genera and are morphologically similar, often exhibiting overlapping sizes of conidia or ascospores. As a result, it is relatively easy to identify specimens at the generic level, but more challenging to distinguish them at the species level (Yang et al. 2020, 2021; Zhu et al. 2023, 2024; Liu et al. 2024). In this study, we present novel findings from Xizang, China, which indicate the potential existence of numerous undescribed species in unexplored or minimally investigated regions worldwide.

*Diaporthealnicola* and *D.amygdali* are here reported to be associated with leaf spot disease of *Alnusnepalensis*, which is a common disease in Linzhi, Xizang, China. Among these pathogens, *D.alnicola* is a novel species and may be the primary pathogen associated with *A.nepalensis*. In contrast, *D.amygdali* is a generalist fungus that infects a wide range of plant hosts, including *Acer* spp., *Camelliasinensis*, *Lithocarpusglabra*, *Prunusdulcis*, *Pr.persica*, *Pr.salicina*, *Pyruspyrifolia*, *Ternstroemiagymnanthera*, *Vacciniumcorymbosum*, and *Vitisvinifera* (Hilário et al. 2021b). This suggests that *D.amygdali* may be a secondary pathogen to *A.nepalensis*. For successful disease management, it will be of paramount importance, albeit challenging, to effectively interrupt the infection cycle of *A.nepalensis* maintained by the occurrence of leaf spots caused by and due to the broad host range of *D.amygdali*. Therefore, future investigations need to focus on identifying other hosts of *D.amygdali* in Linzhi City.

## Supplementary Material

XML Treatment for Diaporthe alnicola

XML Treatment for Diaporthe amygdali

XML Treatment for Diaporthe linzhiensis

## ﻿Additional information

### Author ORCIDs

Jieting Li https://orcid.org/0009-0001-8984-7261

Yi Li https://orcid.org/0009-0004-0656-9799

Jiangrong Li https://orcid.org/0000-0002-6679-5227

Ning Jiang https://orcid.org/0000-0002-9656-8500

### Data availability

All of the data that support the findings of this study are available in the main text.
